# Effect of Cognitive Style on Learning and Retrieval of Navigational Environments

**DOI:** 10.3389/fphar.2017.00496

**Published:** 2017-07-25

**Authors:** Maddalena Boccia, Francesca Vecchione, Laura Piccardi, Cecilia Guariglia

**Affiliations:** ^1^Department of Psychology, “Sapienza” University of Rome Rome, Italy; ^2^Cognitive and Motor Rehabilitation Unit, Fondazione Santa Lucia (IRCCS) Rome, Italy; ^3^Department of Life, Health and Environmental Sciences, L'Aquila University L'Aquila, Italy

**Keywords:** field dependence, human navigation, topographical memory, spatial orientation, patial mental representation, egocentric and allocentric coordinates

## Abstract

Field independence (FI) has been found to correlate with a wide range of cognitive processes requiring cognitive restructuring. Cognitive restructuring, that is going beyond the information given by the setting, is pivotal in creating stable mental representations of the environment, the so-called “cognitive maps,” and it affects visuo-spatial abilities underpinning environmental navigation. Here we evaluated whether FI, by fostering cognitive restructuring of environmental cues on the basis of an internal frame of reference, affects the learning and retrieval of a novel environment. Fifty-four participants were submitted to the Embedded Figure Test (EFT) for assessing their Cognitive Style (CS) and to the Perspective Taking/Spatial Orientation Test (PTSOT) and the Santa Barbara Sense of Direction Scale (SBSOD) for assessing their spatial perspective taking and orientation skills. They were also required to learn a path in a novel, real environment (route learning, RL), to recognize landmarks of this path among distracters (landmark recognition, LR), to order them (landmark ordering, LO) and to draw the learned path on a map (map drawing, MD). Retrieval tasks were performed both immediately after learning (immediate-retrieval) and the day after (24 h-retrieval). Performances on EFT significantly correlated with the time needed to learn the path, with MD (both in the immediate- and in the 24 h- retrievals), results on LR (in 24-retrieval) and performances on PTSOT. Interestingly, we found that gender interacted with CS on RL (time of learning) and MD. Females performed significantly worse than males only if they were classified as FD, but did not differ from males if they were classified as FI. These results suggest that CS affects learning and retrieval of navigational environment, especially when a map-like representation is required. We propose that CS may be pivotal in forming the cognitive map of the environment, likely due to the higher ability of FI individuals in restructuring environmental cues in a global and flexible long-term representation of the environment.

## Introduction

The “environmental space” has been proposed as the portion of the space that can be inspected and learned through considerable movement, that is actually navigating across buildings and neighbors (Wolbers and Wiener, 2014), while the “vista space” concerns the portion of the space that can be visually inspected and learnt from a single location or with little movements (Wolbers and Wiener, 2014). To successfully orientate within the environmental space, individuals rely on at least two sources of information (Wolbers and Hegarty, 2010). The first one is the online representation of the space, which consists of information about the current position and its spatial updating, the egocentric self-to-objects relations and distances, the allocentric relations among environmental objects and the route progression. The second one is the offline representation of the environment, which includes the topographical knowledge that allows for building up a stable internal representation of the environmental space, namely the “cognitive map” (Tolman, 1948). Thanks to the topographical knowledge, individuals can imagine what lies beyond the current vista space and, thus, are able to successfully plan the best route toward their navigational goal.

Three different types of mental representations of the environment, namely the Landmark, Route and Survey representations, have been described (Siegel and White, 1975). *Landmark representation* roughly corresponds to the figurative memory of environmental objects; by using this type of knowledge, individuals are able to “beacon” toward salient landmarks within the vista space. *Route representation* consists of the memory of the path that connects different landmarks in the environmental space, organized on the basis of an egocentric frame of reference; by using this type of knowledge individuals are able to reach a not visible navigational goal by recalling the sequence of landmarks, directions and distances along the path connecting the starting point and the goal. *Survey representation* roughly corresponds to a map-like representation of the environmental space, which implies the encoding of directions and distances between landmarks regardless of the individual's position, that is the use of an allocentric frame of reference; by using this type of knowledge individuals are able to reach a navigational goal planning, novel routes and detours. The organization of topographical knowledge across the three mental representations of the environmental space is cumulative, and hierarchical, with high-level stages encompassing features of the lower stages and being mandatory the acquisition of lower-level stages for higher-level stages (Siegel and White, 1975). Interestingly, the functional activation within the brain network involved in spatial navigation, specifically the retrosplenial cortex (RSC) and parahippocampal gyrus (PHG) (see Boccia et al., 2014 for a review), shows an interaction with the acquisition stage of the topographical knowledge and its format. In particular, both RSC and PHG are activated by the visual scanning of the vista space during the first stage of the acquisition, but also by the mental representation of the position of a non-visible landmark when topographical knowledge has been fully acquired (i.e., after 5 days of spatial training; Boccia et al., 2016a). This is consistent with the idea that well-known environments are represented in a survey format allowing to take into account portions of the environment that are not in the direct view (Siegel and White, 1975).

Levels of navigational skills are greatly variable in humankind and this variability has been shown to correspond to neuroanatomical differences, since *good* navigators show higher gray matter volume in the right hippocampus (Wegman et al., 2014) and higher functional connectivity between the posterior hippocampus and retrosplenial complex (Sulpizio et al., 2016) as compared with *poor* navigators.

Several factors have been proposed to affect individual differences (Wolbers and Hegarty, 2010). First of all, direct correlation among some spatial abilities, such as mental rotation, left-right discrimination (Moffat et al., 1998) and perspective-taking (Kozhevnikov and Hegarty, 2001; Hegarty and Waller, 2004), and navigational skills have been repeatedly shown. Second, levels of navigational ability seem be depending on the strategy individuals adopt for orienteering. Indeed some individuals prefer to orientate themselves by using a landmark strategy, some prefer to use a route strategy and some others a survey strategy (Pazzaglia et al., 2000) and the type of strategy/representation used affect the proficiency in navigational tasks, with the worst performances in individuals adopting the landmark strategy and best performances in those adopting the survey strategy. The preference for one type of strategies has been hypothesized to be affected by Gender. Indeed, females are more prone to adopt a landmark strategy and males a survey one (Nori et al., 2009); males are more proficient than females in survey tasks (Coluccia and Louse, 2004), with females performing worse in locating environmental features on a map of a familiar environment (the University campus; Mcguiness and Sparks, 1983). Interestingly, performances are leveled-off when females are provided with the survey map of the path they are required to learn (Montello et al., 1999), or when they may study a map or explore an environment without time limits (Piccardi et al., 2011a,c). Generally males outperformed females in acquiring new spatial knowledge from direct exposure (Montello et al., 1999), but when participants are matched for preferred strategy (i.e., Route or Survey strategy) no gender effect is observed in navigational skills (Nori et al., 2006; Nori and Piccardi, 2011, 2015; Piccardi et al., 2016).

Field Dependent/Field Independent Cognitive Style (hereafter called CS) has been recently found to affect spatial ability underlining navigational skills and individuals' predispositions toward different navigational styles (Boccia et al., 2016b, 2017a). CS has been proposed as the information processing style that characterizes the way an individual analyzes and organizes the world (Witkin, 1977). Specifically, Field-independent individuals (hereafter FI) rely on an internal frame of reference in processing and organizing environmental information and are not susceptible to deceptive environmental cues. Otherwise, field-dependent individuals (hereafter FD) rely on an external frame of reference and are susceptible to deceptive cues when identifying known elements in unknown settings. CS is usually assessed by using tasks requiring participants to detect embedded simple pictures in complex configurations, such as the Embedded Figures Test (EFT; Witkin et al., 1971), or the Rod and Frame Illusion, requiring participants to align to the vertical midline a rod in the presence of a tilted surrounding frame (Witkin et al., 1954). Both of these tasks are usually performed better by FI. CS affects a wide range of cognitive skills and tasks, especially those requiring to go beyond the information given by the setting, that is tasks requiring cognitive restructuring (Witkin, 1977). Examples of cognitive restructuring are *disembedding* and *perspectivism*. The former refers to the ability to extract salient information from the surrounding field, whereas the latter refers to the ability to recognize and to adopt the perspective of another person (Witkin, 1977). Both these processing may be considered fundamental in the processing of topographical cues. Indeed, navigating within the environmental space requires processing and “restructuring” a huge amount of information in order to create and organize a stable mental representation of the environment. Recently, CS has been found to predict performances on spatial skills underpinning navigation, such as mental rotation and egocentric perspective taking (Boccia et al., 2016b): FI performed better than FD on both tasks. Furthermore, CS predicted individual's preferred navigational strategy, with FI more prone to prefer survey strategy than FD (Boccia et al., 2017a), regardless the gender.

Here we aimed to assess whether CS affected the acquisition of new topographical knowledge and its retrieval. With this aim, participants underwent tasks aimed at assessing the acquisition and the organization of new spatial knowledge from the direct exposure to the environment, that is (1) to learn and retrieve a path within a real environment, (2) to recognize landmarks they faced along the path and (3) to order them, (4) to trace the path on a map; the retrieval tasks (i.e., path retrieving, landmark recognition and ordering, and map drawing) were performed both immediately and after 24 h. They were also assessed for the CS by using the Embedded Figure Test (EFT). We hypothesized that CS predicted the acquisition of new spatial knowledge, especially when it has been requested to reorganize the acquired information into the map (i.e., map drawing task). Corollary, we also assessed a possible Gender-by-CS interaction. We expected that males outperformed females only among FD and that, with equal level of field dependence, gender differences would disappear.

## Materials and methods

### Participants

Fifty-Four healthy college students (mean age 24.70 ± 2.07; 28 females, *t*_(52)_ = 0.04; *p* = 0.97) took part in this study. None of the participants had a history of neurological or psychiatric disease, which was confirmed during an informal interview carried out before the test phase. All participants have normal or corrected-to-normal (soft contact lenses or glasses) vision. Moreover, all participants performed the Familiarity and Spatial Cognitive Style Scale (FSCS) (Piccardi et al., 2011b) which includes 22 self-referential statements about various aspects of environmental spatial cognition. The FSCS was used to exclude participants with self-declared topographical orientation disorders. None of the participants showed the presence of navigational deficits or developmental topographical disorientation (Iaria et al., 2005, 2009; Bianchini et al., 2010).

All participants signed a consent form before the study began. This study was approved by the local ethics committee of I.R.C.C.S. Santa Lucia Foundation, in agreement with the Declaration of Helsinki.

### Assessing field independence: the embedded figure test

As briefly reported above, the individual's predisposition toward the FD or the FI (i.e., Cognitive Style) has been classically assessed by tasks requiring participants to detect embedded simple figures in complex configurations, such as the (EFT; Witkin et al., 1971) and the hidden figures test (Ekstrom et al., 1976). Generally, FI individuals, by ignoring contextual information, are more able at detecting the embedded figures than FD individuals, who are more affected by the contextual (almost deceptive) information of the complex configurations and are less able at detecting the embedded figures in the whole configurations (Witkin, 1977; Witkin et al., 1977; Walter and Dassonville, 2011).

We used the EFT (Witkin et al., 1971) to assess individual's predisposition toward FI/FD. Participants were tested individually in a quiet, well-lit room. The participants were explained that they had to find a simple geometric shape within a larger complex figure. The experimenter presented the larger complex colored figures one-by-one for 15 s, on a 12.9 × 7.7 cm card. During the presentation time, participants were asked to orally describe the figure. After this period, the experimenter removed the complex figure and presented the simple black/white figure, for 10 s, on a card of the same size of the complex figure. Then, he/she removed the simple figure and presented once again the complex figure, asking participants to find that simple figure within the complex one. Participants were also required to advise the experimenter as soon as they found the embedded simple figure and then to trace it by using a stylus. The experimenter clocked the time. When participants said that he/she has find the simple figure, the experimenter annotated the time passed (timing): if the response was correct, that time represented the response time; otherwise, if the response was wrong, the experimenter continued to clock the time until participant produced the correct response or until 180 s had been passed. The total time was computed by summing the response time on each item. The total time was then divided by the number of items (12) to compute the overall time averaged across items. Averaged times (EFT scores) were used as the measure of the individual's Cognitive Style (CS), with lower times indicating individuals with higher predisposition toward the FI.

### Navigational abilities in experimental environment

#### Spatial perspective taking

The PTSOT is a spatial orientation task, proposed by Kozhevnikov and Hegarty (2001). It is a paper-and-pencil test frequently used to assess egocentric perspective taking (Hegarty and Waller, 2004). The PTSOT is composed of 12 trials. In each trial, an array of 7 objects is drawn on the top half of a 210 × 297 mm sheet. On each trial, participants are asked to imagine being placed at the position of one object in the array (imagined position), facing another object (heading direction) and to indicate the direction to a third object (the target object). A circle is depicted on the bottom half of the page. The imagined position is drawn in the center of the circle, while the heading direction is drawn as an arrow pointing vertically up. Participants are asked to draw an arrow from the center of the circle (imagined position) to indicate the direction to the target object (Kozhevnikov and Hegarty, 2001). For each participant and trial, the absolute deviation in degrees between the individual's response and the correct direction to the target was computed (Kozhevnikov and Hegarty, 2001; Hegarty and Waller, 2004). For each participant, the total score is the average deviation across all trials (Kozhevnikov and Hegarty, 2001; Hegarty and Waller, 2004). Lower scores (i.e., lower deviation from the correct direction) corresponded to better performances.

#### Self-reported assessment of spatial orientation ability

The Santa Barbara Sense of Direction Scale (SBSOD) is a self-report questionnaire (Hegarty et al., 2002) that has been shown to strongly correlate with actual navigation ability (Janzen et al., 2008; Wegman et al., 2014). Following Hegarty et al. (2002), after reverse scoring, the sum of the scores for all of the items was calculated and then divided by the number of items (i.e., 15) to compute the average score across items for each participant. The SBSOD scores ranged between 1 and 7, with higher scores corresponding to a better-perceived sense of direction.

### Navigational abilities in ecological environment

**Figure 1:**
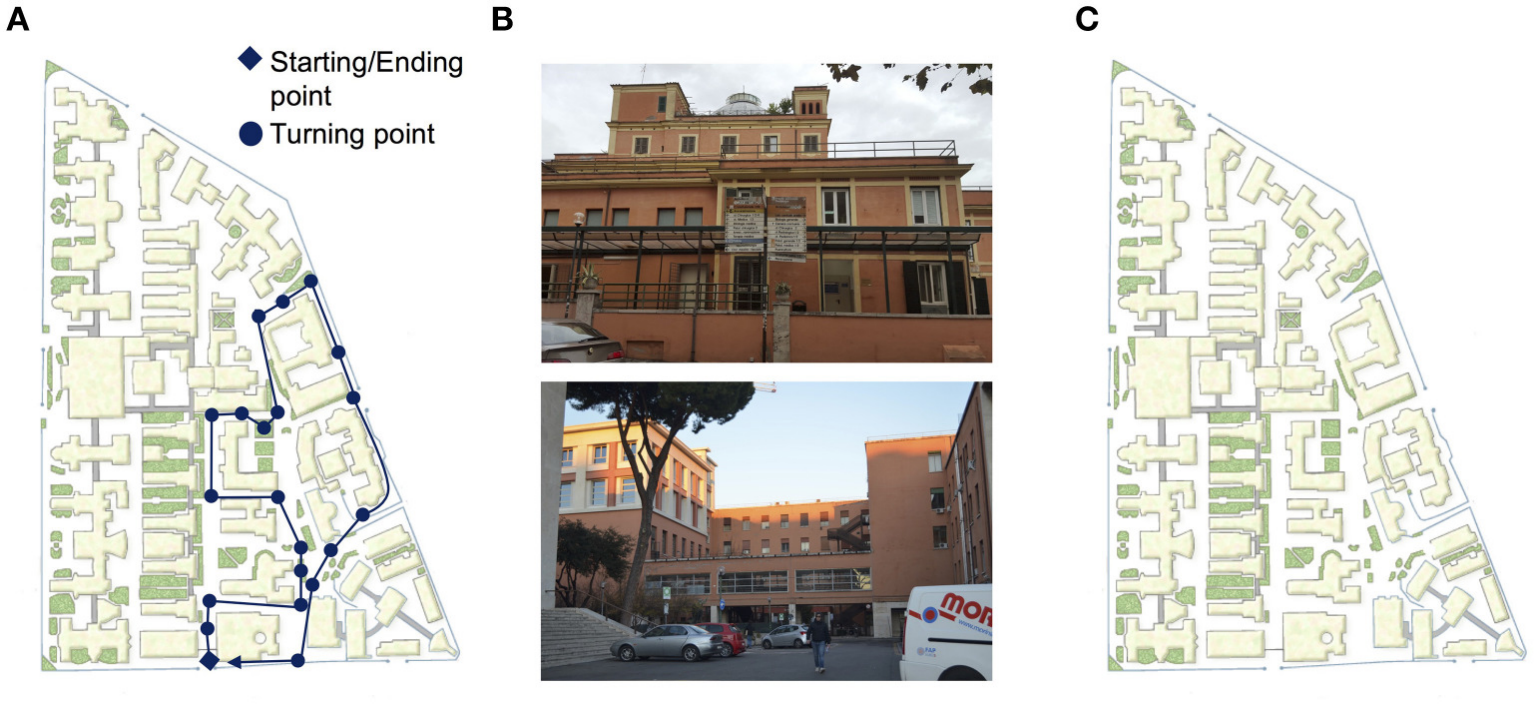
Tasks assessing navigational abilities in ecological environment. **(A)** Path within “Umberto I” general hospital. Circles and rhombus indicate the turning points and the starting/ending point, respectively. **(B)** Example of landmark (on the top of the panel) and distracter (on the bottom of the panel) used in landmark recognition task. **(C)** Sketch map used during map drawing task.

Participants were requested to learn an out-door path within “Umberto I” general hospital campus in Rome (Figure 1A). The path encompassed 20 turning points, balanced across left (*N* = 5), right (*N* = 7), and straight (*N* = 8). Eight landmarks (and 8 distracters) have been selected for the landmark recognition task (see the description below).

#### Route learning

The experimenter showed the path (Figure 1A) and asked the participant to pay attention to each landmark and turn across the path. At the end of the path (immediate-recall), the experimenter drove the participant at the starting point and asked him/her to *retrieve the path* (route learning, RL). The experimenter corrected the participants if they took the wrong decision on the turning points and, once they completed the whole path, he/she asked the participants to retrieve the path again (from the starting point) until they correctly performed the whole path. Participants who learnt the path on the first attempt were not required to retrieve the path again. All participants correctly retrieve the whole path until the second attempt. The learning score was calculated by attributing one point for each turn correctly performed, until the criterion was reached (i.e., all turns correctly performed); then it was added to the score corresponding to correct performance of the remaining attempts (up to the 2nd; maximum score: 40). For example, if the participant correctly retrieved the path by the first attempt, he/she obtained 20 on the first attempt and 20 on the second attempt, thus his/her score on RL was 40. Otherwise, if the participants correctly retrieved 18 out of the 20 turns of the path on the first attempt and all the turns on the second one, he/she obtained 18 on the first attempt and 20 to the second one. Thus, his/her score on RL was 38. Time needed to reproduce the path (seconds) was also registered and used for further analyses.

#### Landmark recognition and ordering

After the RL, the participants were presented with eight pictures of landmarks encountered along the way (e.g., a building) interspersed with eight pictures of distracters (e.g., a building similar to the actual landmark; Figure 1B). The participants had to indicate for each picture whether it represented the landmark encountered along the path or not (landmark recognition, LR; maximum score: 16). Then, they were asked to order the landmarks they identified (landmark ordering, LO; maximum score: 8).

#### Map drawing

The participants were asked to draw the path on the sketch map of the “Umberto I” general hospital (map drawing, MD; Figure 1C). The score was calculated by attributing one point for each turn that had been correctly drawn by the participants (maximum score: 20).

#### Delayed recall

One day after (about 24 h later, 24 h-retrieval), participants were requested (1) to reproduce the path they learned without additional demonstration (delayed route recall, dRR), (2) to recognize and (3) to order the landmarks (respectively, delayed landmark recognition, dLR, and delayed landmark ordering, dLO) and (4) to draw the path on the sketch map of the general hospital (delayed map drawing, dMD).

### Statistical analyses

Statistical analyses were performed by using SPSS. First, we computed Pearson's correlation coefficients among different tasks and EFT. Level of significance was set at *p* = 0.05. Thus, we performed linear regression analyses with EFT scores as predictor and score on navigational tasks (which result to be significantly correlated with EFT) as dependent variables. Significant *p* has been estimated by applying Bonferroni's correction for multiple comparisons.

Among these tasks, we further assessed whether gender interacted with CS in determining individuals' performances. To this aim, we calculated quartiles on EFT scores. Thus, we classified participants of the first quartile (i.e., fastest individuals) as Field Independent (FI; *N* = 13) and those of the fourth quartile (i.e., slowest individuals) as Field Dependent (FD; *N* = 13). Individuals who fell within the second and third quartiles were excluded from further analysis. Males and females were equally distributed across FI (6 females and 7 males) and FD (5 females and 8 males) individuals (Chi-squared = 0.158; *p* = 0.691). Thus, we performed a Multivariate Analysis of Variance (MANOVA), by entering Gender and CS as independent variables and performances on PTSOT, time of RL, MD, dLR, and dMD as dependent variables. Level of significance was set at *p* = 0.05 and Bonferroni's correction for multiple comparisons has been applied on pairwise comparisons.

## Results

The Pearson's correlation among different tasks and EFT are reported in Table 1. To assess whether CS predicted performances on navigational tasks we performed linear regression analyses with EFT scores as predictor and score on navigational tasks (which have been found to be correlated with EFT) as dependent variable. We found that time of RL (β = 0.284; *t* = 2.135; *p* = 0.038), MD (β = −0.375; *t* = −2.916; *p* = 0.005), dLR (β = −0.342; *t* = −2.625; *p* = 0.011) and dMD (β = −0.414; *t* = −3.283; *p* = 0.002), as well as performances on PTSOT (β = 0.436; *t* = 3.494; *p* = 0.001), were significantly predicted by EFT scores. Effects on MD, dMD, and PTSOT were still significant when Bonferroni's correction for multiple comparisons was applied (significance level *p* = 0.01).

**Table 1:** Pearson's correlations.

		**EFT scores**	**PTSOT**	**SBSOD**	**RL**	**Time for RL**	**LR**	**LO**	**MD**	**dRR**	**dLR**	**dLO**	**dMD**
EFT scores	r	1	*0.436*	0.031	−0.162	*0.284*	−0.141	0.011	−*0.375*	0.098	−*0.342*	−0.158	−*0.414*
	*p*		*0.001*	0.824	0.242	*0.038*	0.308	0.939	*0.005*	0.479	*0.011*	0.255	*0.002*
PTSOT	r		1	−0.044	0.034	−0.010	0.008	−0.133	*−0.346*	−0.004	−0.090	−0.112	*−0.392*
	*p*			0.754	0.807	0.940	0.957	0.338	*0.010*	0.976	0.517	0.419	*0.003*
SBSOD	r			1	*0.351*	*−0.325*	−0.043	0.096	0.088	−0.018	−0.079	0.149	0.210
	*p*				*0.009*	*0.017*	0.760	0.491	0.526	0.896	0.572	0.281	0.128
RL	r				1	*−0.872*	0.180	0.108	0.151	0.006	−0.001	0.118	0.165
	*p*					*0.000*	0.194	0.437	0.277	0.968	0.995	0.397	0.233
Time for RL	r					1	−0.088	−0.129	−0.117	0.235	−0.006	−0.168	−0.110
	*p*						0.526	0.353	0.399	0.088	0.968	0.223	0.430
LR	r						1	*0.361*	0.155	0.060	*0.662*	0.237	0.104
	*p*							*0.007*	0.264	0.667	*0.000*	0.085	0.453
LO	r							1	0.247	−0.095	*0.366*	*0.472*	0.102
	*p*								0.071	0.494	*0.007*	*0.000*	0.463
MD	r								1	0.195	0.227	0.126	*0.748*
	*p*									0.158	0.098	0.364	*0.000*
dRR	r									1	−0.114	−0.266	0.138
	*p*										0.414	0.052	0.321
dLR	r										1	*0.571*	0.219
	*p*											*0.000*	0.111
dLO	r											1	0.183
	*p*												0.186
dMD	r												1
	*p*												

*r, Pearson's correlation coefficient; p, p-value; PTSOT, Perspective Taking/Spatial Orientation Test; SBSOD, Santa Barbara Sense of Direction Scale; RL, Route Learning; LR, Landmark Recognition; LO, Landmark Ordering; MD, Map Drawing; dRR, Delayed Route Recall; dLR, Delayed Landmark recognition; dLO, Delayed Landmark Ordering; dMD, Delayed Map Drawing. Italic values indicate the r and p statistically significant*.

**Figure 2:**
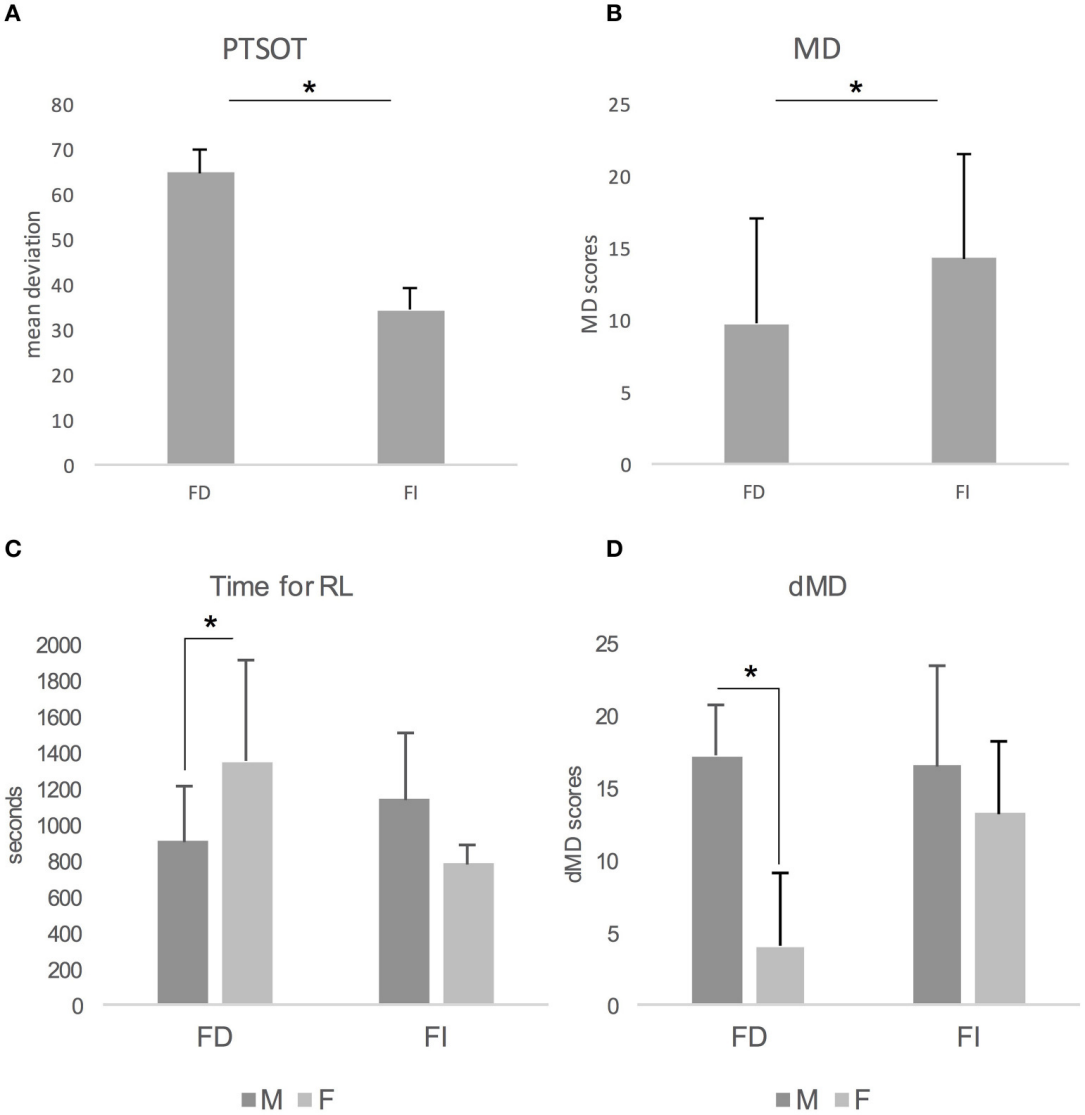
Effect of Cognitive Style on PTSOT **(A)** and Map Drawing task **(B)**. Gender by Cognitive Style interaction on time needed to learn the path **(C)** and on delayed map drawing **(D)**. Bars depict mean and standard deviation of performances. FI, Field Independent; FD, Field Dependent; M, Males; F, Females; PTSOT, Perspective Taking/Spatial Orientation Test; RL, route learning; MD, map drawing; dMD, delayed map drawing.

Concerning the results of MANOVA, we found a main effect of Gender on MD [*F*_(1, 22)_ = 6.432; *p* < 0.05; Partial Eta Squared = 0.226; Observed Power = 0.679] and dMD [*F*_(1, 22)_ = 14.699; *p* < 0.01; Partial Eta Squared = 0.401; Observed Power = 0.956]. In both cases, males (*MD*: *M* = 14.530, *SD* = 6.334; *dMD*: *M* = 16.800, *SD* = 5.226) outperformed females (*MD*: *M* = 8.450, *SD* = 7.647; *dMD*: *M* = 9.00, *SD* = 6.943). We also found a main effect of CS on PTSOT [*F*_(1, 22)_ = 9.549; *p* < 0.01; Partial Eta Squared = 0.303; Observed Power = 0.840; Figure 2A] and MD [*F*_(1, 22)_ = 4.549; *p* < 0.05; Partial Eta Squared = 0.171; Observed Power = 0.532; Figure 2B]. In both cases, FI performed better than FD. Actually, FI individuals showed lower deviation from correct response on PTSOT (*M* = 34.282, *SD* = 19.477) than FD individuals (*M* = 64.526, *SD* = 33.393). FI also performed better (*M* = 14.230, *SD* = 7.178) than FD (*M* = 9.690, *SD* = 7.239) on MD. Interestingly, we found that Gender and CS interacted on Time for RL [*F*_(1, 22)_ = 8.057; *p* < 0.05; Partial Eta Squared = 0.268; Observed Power = 0.774] and dMD [*F*_(1, 22)_ = 5.325; *p* < 0.05; Partial Eta Squared = 0.195; Observed Power = 0.597]. Concerning Time for RL, females were slower than males only in the FD group of participants (*p* = 0.040, Bonferroni's correction for multiple comparisons; Figure 2C). Females performed worse than males only in FD group (*p* < 0.001, Bonferroni's correction for multiple comparisons) also on dMD (Figure 2D).

## Discussion

Here we found that Field dependence/independence Cognitive Style (CS) affects performance on several navigational tasks, that is map drawing task (both immediate and delayed recall) and PTSOT. FI individuals performed better than FD ones in map drawing task and PTSOT. Corollary, we found that gender interacted with CS in time for route learning and delayed map drawing task, and gender differences were detectable just in FD individuals.

CS is a pervasive characteristic of individuals' perceptual and intellectual functioning which cannot be affected by experience or learning (Witkin, 1967, 1977). As reported in the introduction, CS affects a wide range of abilities, such as mental rotation and egocentric perspective taking, especially when, as often happens in navigation, cognitive restructuring is required (Boccia et al., 2016b). Indeed, to successfully orientate within the environmental space, environmental inputs and knowledge need to be continuously restructured; individuals have to continuously restructure information given by the context (i.e., the current vista space and the environmental space), to online update both spatial cues in the environment and their current relative (one in respect of the other) and absolute (in respect of allocentric references) positions, to process spatial computations of Euclidean/metric environmental features (for example, distances), to translate the egocentric representations of the environment into allocentric ones (and vice versa), to monitor the route progression and to plan novel routes, to develop the online representations of the environment and to retrieve the offline (i.e., previously acquired) topographical knowledge (Wolbers and Hegarty, 2010).

Here we assessed the effects of CS on spatial navigation within a real environmental space, by testing the acquisition (*route learning task*) and retrieval (*delayed route recall*) of a novel open-field environment; also, landmark recognition and the knowledge of their relative spatial positions (*landmark recognition* and *landmark ordering*) were evaluated. Cognitive restructuring was fostered by asking participants to transform route learning into a 2D map-like representation (*map drawing*).

In agreement with our hypothesis, FI individuals were more able than FD in the map drawing task and in reporting learned path into a map (both immediately and after 24 h). Even if no significant difference was observed, it should be noticed that regression showed a trend in FI participants toward less time required for learning the path (i.e., total time required to learn the path) and better ability to recognize landmarks after 24 h. Indeed, all participants, independently from their CS, were able to learn the path within the second attempt, to recognize all the landmarks and to correctly retrieve the order in which they had met them along the route. Instead, CS strongly affects the map-drawing skill, which is significantly predicted by performances on EFT. These results suggest that environmental navigation skills are not generally affected by CS, which plays a very specific role on abilities, such as those involved in map drawing, related to the “creation” of a flexible mental representation of the environmental space.

This interpretation is supported by a previous study showing that FI individuals are more likely to prefer and adopt a survey strategy, while FD ones are very likely to prefer and adopt a landmark strategy (Boccia et al., 2017a). These results allow to hypothesize that the evolved navigational strategy, namely the survey strategy, may be easily accessible to individuals with a FI CS, but not to those with a FD CS. As a consequence, FI individuals should show better performances whenever the vista representation alone and route/landmark strategies are not sufficient for achieving a navigational goal or whenever a survey/allocentric representation of the environmental space is required. Some results support this hypothesis. Indeed, Boccia et al. (2017a) found FI individuals were more proficient in performing the “survey” tasks, that is tasks requiring participants to use an abstract, internal, object-based representation of the space, which almost corresponds to a “bird's-eye viewpoint” (i.e., survey representation) (Nori and Giusberti, 2006) and in present study FI were significantly better in both the map drawing tasks. The ability to represent in a map what has been acquired while exploring a novel environment depends on the ability to develop a cognitive map, that is an allocentric, survey representation of the environment (Iaria et al., 2007). Following some current navigational models, allocentric representations are developed by familiarization with the environment (Siegel and White, 1975; Montello, 1998) and higher is the familiarity better is the allocentric representation. In present study, however, the better performance on map drawing in FI individuals cannot be explained as an effect of familiarity, since their time of familiarization with the novel environment they had never been exposed before did not differed from that of FD. Thus, differences in performance should depend on differences in the way the navigational features are processed depending on individuals' CS. FI individuals are able to process navigational information in an evolved allocentric representation in a quicker and more effective way than FD; the effect of CS on the ability to develop, store and retrieve allocentric representations is stable across time, since, CS predicted individual's performance in map drawing not only soon after the end of the learning (immediate-retrieval), but also in the retest after 24 h (24 h- retrieval). Present study does not allow to understand if an increased familiarization would improve FD's performances, since in our paradigm a fixed learning experience was provided and future studies are necessary for understanding if individuals with very high levels of FD are able to develop an evolved allocentric representation if provided of extensive familiarization. However, present results clearly underline the advantage of FI in developing map representation even in absence of differences from FD in learning novel routes in a previously unknown place.

Why FI individuals are more proficient in developing the survey/allocentric representations? To perform the map drawing tasks, individuals had to transform the environmental information coded during an egocentric experience of the “vista space” (Wolbers and Wiener, 2014) into an allocentric, map-like representation of the “environmental space” (Wolbers and Hegarty, 2010; Wolbers and Wiener, 2014), in which the positions of the landmarks, as well as the relative distances between landmarks, have to be represented regardless of the individual's position. Thus, this task fostered the individual's ability to “restructure” the egocentrically acquired route knowledge into an abstract, survey representation of the environmental space. As stated above, FI individuals usually rely on an internal frame of reference in restructuring the environmental information. Their higher ability in map drawing task may be *generally* due to the fact that they rely on the internal frame of reference to proficiently restructure the “route” knowledge about a given environment into the corresponding “survey” representation. In this light, the survey representation of the environmental space should rise from an internally driven reorganization of the egocentrically acquired spatial knowledge of neighboring vista spaces. Another characteristic of FI, that is *perspectivism*, should also play a role in the better performance of FI individuals. As reported above, field-independent individuals are usually more able than field-dependent individuals in adopting the perspective of another person. This capability to “look from another point of view” could ease the spatial computation allowing the translation of egocentrically acquired route knowledge into map-like, survey representation. This possible role of *perspectivism* in developing survey representations is consistent with finding that map drawing is significantly correlated both with CS and performances on egocentric perspective taking task (see Table 1).

As reported above, CS only marginally affected the time for learning and the delayed landmark recognition tasks without no significant group differences between FI and FD individuals. Also, we did not find any effect of the CS on Route Learning and Landmark Recognition (immediate-recall). Both these tasks do not require cognitive restructuring, since they tap on the memory for the path and landmark in the vista space, as it happens for Map-drawing task, but also they do not involve perspectivism, since no change of the point of view is required for deciding if a landmark was on the route and if it follows or not a another given landmark.

Taken together, our data suggest that FI/FD CS have specific effects only on some of the navigational skills, and in particular only on the ability to build up complex representations of the environmental space starting from the knowledge acquired form the vista space, perhaps affecting also the ability to translate the format of the environmental knowledge from one type of frame of reference to another (for example from the egocentric to the allocentric frame of reference and vice versa). Following this interpretation, the effects of CS should be evident in all the tasks requiring a reorganization of environmental knowledge, even those in which no direct spatial translation is required, such as, for example, when a detour from a familiar route is required to cope with a blocked-route. In this case the task forces individuals to “restructure” previous knowledge to solve the navigational “request” (Hirshhorn et al., 2012) moving form a vista-space representation to an environment-space one.

Present results confirm those about egocentric perspective taking task obtained by Boccia et al. (2016b), who showed that CS predicts performances on PTSOT. In this previous study, CS was assessed by using the Group Embedded Figure Test (GEFT), a test differing from the EFT used in present study in different aspects. Indeed, despite using the same stimuli, the two tests differ both in the administration (the GEFT is administered to groups of participants simultaneously, whereas the EFT is administered individually) and in behavioral indexes used for scoring, since the GEFT uses the accuracy as the index of CS, while the EFT uses the response time. Results of present study, thus, not only confirm the previous one, but also offer a convergent evidence about the relation between the egocentric perspective taking and the CS.

As a corollary aim, we assessed whether CS interacted with Gender in determining navigational skills. As a group, females performed worse than males on map-drawing, both immediately and after 24 h. These results are consistent with previous literature about gender differences in navigational skills (Coluccia and Louse, 2004). However, we also found that gender interacts with CS on the time needed to learn the path (i.e., time for route learning) and delayed map-drawing (interaction has been not detected for immediate map-drawing). Females performed worse than males only if they are FD individuals, while no gender difference was present in the group of FI individuals. This result mirrors that of a previous study finding that when men and women were matched for their spatial style they do not show differences in their spatial orientation ability (Nori and Giusberti, 2003, 2006). Also, the present result points toward a pivotal role of CS in gender-related differences in environmental navigation, prompting to put attention to this dimension in future investigations about gender differences. Indeed, the observation that only field dependent females show worst performances than males suggests the possibility that contrasting results about gender differences in navigational and topographical skills can be due to the presence of different percentage of FI and FD women in different studies, so that studies in which a higher percentage of FI is included in the female group failed in finding gender differences that were instead present in the studies in which the female group included a higher percentage of FD.

Interestingly, even if CS predicted performance on several navigational tasks (see above for detailed discussion about the effect of CS on map-drawing task and egocentric perspective taking), there is no significant effect for self-reported navigational skills. This is consistent with a previous investigations (Boccia et al., 2016b), confirming that individuals' meta-cognition about their own spatial abilities do not always correspond to their actual capability. Also, here we found that self-reported navigational skills on SBSOD were significantly correlated with performances and time of route learning, but they were not correlated with performances on the map-drawing task, which is the “navigational skills” mainly affected by CS.

The present findings may have some importance in the field of developmental and acquired topographical disorientation. Topographical disorientation has been described as a consequence of acquired brain damage (Aguirre and Esposito, 1999), congenital malformation (Iaria et al., 2005), normal and pathological aging (Boccia et al., 2016c; Nemmi et al., 2017) as well as in individuals who never develop such an ability (Developmental Topographical Disorientation; DTE) (Iaria et al., 2009, 2014; Bianchini et al., 2010, 2014; Iaria and Barton, 2010; Iaria, 2013; Palermo et al., 2014a,b; Nemmi et al., 2015). Further studies should investigate whether and how CS affects development of topographical orientation ability and/or loss of such an ability due to acquired brain damage or cognitive decline in pathological and normal aging.

The neural mechanisms underlying the relation between CS and environmental navigation have never been explored. Neuroimaging investigations about the neural underpinnings of field-independence/field-dependence suggest a pivotal role of the superior parietal lobe (Walter and Dassonville, 2011; Lester and Dassonville, 2014), an area that has been repeatedly found to be engaged in spatial navigation (Boccia et al., 2014). Whether and how the parietal lobe is involved in the relationship between CS and spatial navigation is a fascinating issue that further studies need to address. However, some data in literature support this hypothesis. Following the model proposed by Byrne and colleagues (Byrne et al., 2007), in the parietal lobe there should be the neural substrate of the “egocentric parietal window” that allows the egocentrically coded information in the parietal lobe to access the allocentrically stored information in the medial temporal lobe, in service of the mental imagery and the spatial navigation. Neuroimaging evidence in both humans (Boccia et al., 2016d, 2017b) and primates (Kravitz et al., 2011) seems to support the model.

In conclusion, the present results confirm that CS affects environmental navigation, especially when a map-like representation is required. Thus, FI is pivotal to restructure the environmental information in a global and flexible long-term representation of the environment, namely the cognitive map, as well as in easing the changes of perspective which allow individuals to re-orient and recognize places from a point of view different from the familiar one.

## Ethics statement

This study was carried out in agreement with the Declaration of Helsinki with written informed consent from all subjects. The protocol was approved by the local ethical committee of Santa Lucia Foundation.

## Author contributions

All the authors conceived the study. MB and FV prepared the stimuli and the tasks. FV collected the data and MB analyzed the data. MB wrote a first draft of the manuscript. All the authors contributed to the revision of this first draft and the discussion of the results.

### Conflict of interest statement

The authors declare that the research was conducted in the absence of any commercial or financial relationships that could be construed as a potential conflict of interest.
